# Surgical site infections rates among patients with craniotomy. Results of a prospective surveillance program in a university teaching hospital

**DOI:** 10.1186/2047-2994-4-S1-P78

**Published:** 2015-06-16

**Authors:** P Ciercoles, A Soriano, E Shaw, A Gabarrós, I Pelegrin, C Cabellos, D Garcia, M Pujol

**Affiliations:** 1 Infection Control, Hospital Universitari de Bellvitge, Hospital de Llobregat, Barcelona, Spain; 2 Neurosurgery, Hospital Universitari de Bellvitge, Hospital de Llobregat, Barcelona, Spain; 3 Infectious Diseases, Hospital Universitari de Bellvitge, Hospital de Llobregat, Barcelona, Spain; 4 Microbiology, Hospital Universitari de Bellvitge, Hospital de Llobregat, Barcelona, Spain

## Introduction

Surgical site infections (SSI) after neurosurgery have serious clinical consequences and increase costs. Surveillance of SSI after craniotomy is not carried out enough.

## Objectives

To describe the results of a SSI surveillance program among patients after craniotomy.

## Methods

Prospective surveillance in a 700-bed university hospital in Barcelona, Spain. The study included all patients admitted for elective or urgent craniotomy from October 2012 to December 2014. All craniotomies followed up within 30 days of surgery or after 1 year if implant inserted.

CDC definitions for SSI were used. Prosthetic implants included plates and screws for cranial osteosynthesis.

## Results

Overall, 469 patients were followed; 54% women, median age of 52 years (range: 18-84). Among them, 70% were elective procedures and 30% urgent or delayed. An implant to secure the bone flap to the skull was used in 426 (91%) patients. SSI developed in 61 patients (13%), organ/space 9%, deep incisional 3.5% and superficial 0.5%. Thirty-three patients (54%) required readmission. Mean days from surgery to infection were 40d. Comparison between patients with and without SSI did not show differences in age, sex, type of surgery (elective or urgent), implant insertion, operation duration, and adequacy of antimicrobial prophylaxis. SSI rates vary depending on ASA score and reason for neurosurgery. Common causative bacteria was Coagulase-negative staphylococci (33%) followed by *Propionibacterium spp* (26%) *Staphylococcus aureus* (21%) and gram negative bacilli (33%). Thirteen out of 61 episodes were polymicrobial.

## Conclusion

Surveillance of craniotomy has allowed us to determine SSI rates and risk factors for SSI. Our results require the implementation of preventive measures.

## Disclosure of interest

None declared.

